# Targeted Curing of All Lysogenic Bacteriophage from *Streptococcus pyogenes* Using a Novel Counter-selection Technique

**DOI:** 10.1371/journal.pone.0146408

**Published:** 2016-01-12

**Authors:** Chad W. Euler, Barbara Juncosa, Patricia A. Ryan, Douglas R. Deutsch, W. Michael McShan, Vincent A. Fischetti

**Affiliations:** 1 Laboratory of Bacterial Pathogenesis and Immunology, The Rockefeller University, NY, NY, 10065, United States of America; 2 Department of Medical Laboratory Sciences, Belfer Research Building, Hunter College, CUNY, New York, NY, 10065, United States of America; 3 Department of Microbiology and Immunology, Weill Cornell Medical College, New York, NY, 10065, United States of America; 4 Department of Pharmaceutical Sciences and Microbiology and Immunology, The University of Oklahoma Health Sciences Center, Oklahoma City, OK, 73117, United States of America; Centers for Disease Control & Prevention, UNITED STATES

## Abstract

*Streptococcus pyogenes* is a human commensal and a bacterial pathogen responsible for a wide variety of human diseases differing in symptoms, severity, and tissue tropism. The completed genome sequences of >37 strains of *S*. *pyogenes*, representing diverse disease-causing serotypes, have been published. The greatest genetic variation among these strains is attributed to numerous integrated prophage and prophage-like elements, encoding several virulence factors. A comparison of isogenic strains, differing in prophage content, would reveal the effects of these elements on streptococcal pathogenesis. However, curing strains of prophage is often difficult and sometimes unattainable. We have applied a novel counter-selection approach to identify rare *S*. *pyogenes* mutants spontaneously cured of select prophage. To accomplish this, we first inserted a two-gene cassette containing a gene for kanamycin resistance (Kan^R^) and the *rpsL* wild-type gene, responsible for dominant streptomycin sensitivity (Sm^S^), into a targeted prophage on the chromosome of a streptomycin resistant (Sm^R^) mutant of *S*. *pyogenes* strain SF370. We then applied antibiotic counter-selection for the re-establishment of the Kan^S^/Sm^R^ phenotype to select for isolates cured of targeted prophage. This methodology allowed for the precise selection of spontaneous phage loss and restoration of the natural phage *attB* attachment sites for all four prophage-like elements in this *S*. *pyogenes* chromosome. Overall, 15 mutants were constructed that encompassed every permutation of phage knockout as well as a mutant strain, named CEM1ΔΦ, completely cured of all bacteriophage elements (a ~10% loss of the genome); the only reported *S*. *pyogenes* strain free of prophage-like elements. We compared CEM1ΔΦ to the WT strain by analyzing differences in secreted DNase activity, as well as lytic and lysogenic potential. These mutant strains should allow for the direct examination of bacteriophage relationships within *S*. *pyogenes* and further elucidate how the presence of prophage may affect overall streptococcal survival, pathogenicity, and evolution.

## Introduction

*Streptococcus pyogenes* (Group A streptococci) is a Gram-positive bacterial pathogen capable of causing a broad spectrum of disease at different sites in the human body. These range from mild infections like pharyngitis and impetigo to more severe invasive infections such as streptococcal toxic shock syndrome and necrotizing fasciitis (reviewed in Cunningham (1)). When inadequately treated, these infections can also lead to the development of post-streptococcal sequelae, such as glomerulonephritis and rheumatic fever [1].

With the advent of more advanced DNA sequencing capabilities and genomic analysis tools, it is now possible to assess the contribution of a particular genotype to the type and severity of a streptococcal infection. In 2001, Ferretti *et al*. reported the first complete genome sequence of an M1 serotype; strain SF370, isolated from a wound infection [2]. The completed genome revealed that this streptococcal strain is poly-lysogenized, and that the integrated bacteriophages (prophages) encode a number of putative virulence factors, suggesting an intimate relationship between prophage and streptococcal pathogenesis [2].

**Fig 1:**
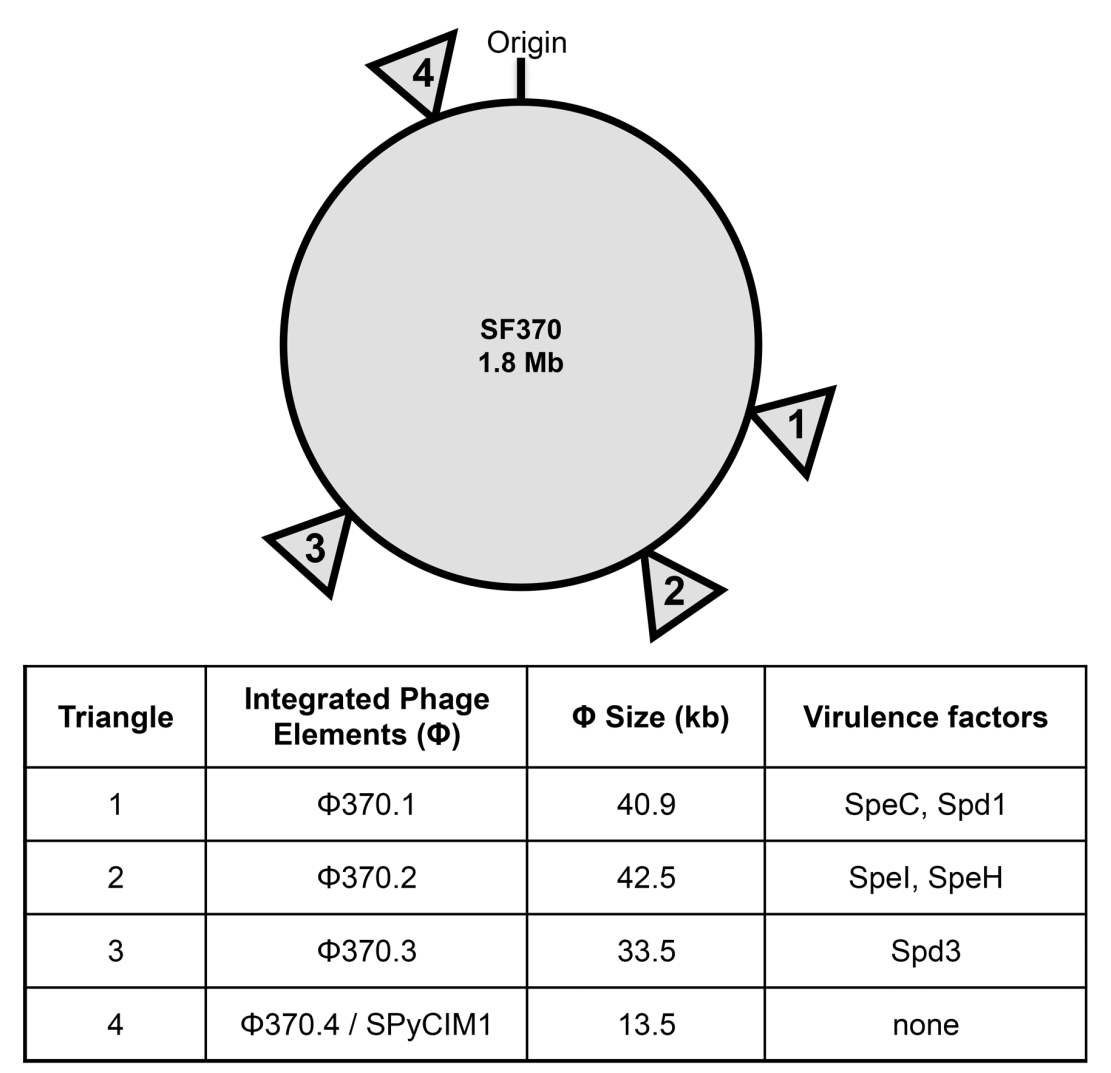
Location of the major bacteriophage elements in the chromosome of SF370. Triangles represent the location of the four main bacteriophage-like elements in the circular genome of *S*. *pyogenes* strain SF370. Numbers in triangles represent the corresponding phage elements with their size and encoded virulence factors identified in the table below. Derived from GenBank nucleotide accession number: AE004092.

The SF370 genome contains many potential mobile genetic elements (MGEs), such as multiple transposons, one integrative and conjugative element (ICE), and four main bacteriophage-like elements (Φ370.1, Φ370.2, Φ370.3 and Φ370.4/SpyCIM1, summarized in Fig 1). Only one prophage (Φ370.1) was previously demonstrated to be inducible into phage particles by mitomycin C treatment, whereas the other three phage-elements are thought to be defective in this capacity, but still encode functional genes [2–4]. Phage Φ370.1 encodes a 41kilobase (kb) genome and contains the *speC* superantigen gene and the *spd1* DNase gene. Phage Φ370.2 is encoded by a 43 kb genome and includes two putative superantigen genes (*speH* and *speI*). Phage Φ370.3, is encoded by a 33 kb genome and harbors another DNase gene (*spd3*).

The 13 kb genome of Φ370.4 contains lysogeny and regulatory modules similar to lambdoid prophages, but lacks identifiable structural, lysis, or virulence genes [2, 3], and has recently been renamed SpyCIM1 (***S****treptococcus* ***py****ogenes* **C**hromosomal **I**sland (SpyCI) M1) [5]. We will use this nomenclature throughout the work presented here, because of its similarity to the phage-related chromosomal islands identified in *Staphylococcus aureus* and other Gram-positive bacteria [6–8]. SpyCIM1 mediates expression of the methyl-directed mismatch repair (MMR) operon through a process of dynamic excision and re-integration from the 5’ end of the *mut*L ORF, and as such has been linked to an increased mutation rate in host bacteria [5, 9].

As of July 2015, the completed chromosomal sequences of approximately 37 other *S*. *pyogenes* genomes have been published and/or deposited into DDBJ, EMBL, and GenBank, and represent diverse M-protein (M) serotypes (e.g., M1, M2, M3, M4, M5, M6, M12, M14 M18, M23, M28, M49, M53, and M59) [2, 10–27]. Although the isolates represent over 13 different M-serotypes recovered from a variety of different streptococcal diseases, body sites, temporal and geographical distributions, the core chromosomal coding sequences of the majority of isolates are 90% similar [28]. The highest degree of genetic variation among strains is due to the numerous phage and phage-like (SpyCI) elements integrated into each of the *S*. *pyogenes* chromosomes [2, 14, 15, 20, 28]. All sequenced *S*. *pyogenes* genomes are poly-lysogenized, containing between one to seven bacteriophage or SpyCI elements. Encoded on these prophage are multiple virulence factors: including superantigens (e.g., SpeH, SpeK, and SpeM), streptodornases or DNases (e.g., Spd1/MF2, Spd4, Sda, and Sdn), phage-encoded, antibiotic resistant cassettes (MefA) [29–31], and phospholipases (Sla) (summarized in [13, 22, 32]).

While comparisons of the published completed genomes show that prophages and prophage-like elements account for most of the sequence differences among various serotypes, in-depth comparative genomic and microarray analyses have confirmed that phage also account for the greatest intra-serotype genetic differences [20, 24, 33, 34]. To that point, a retrospective analysis of the bacteriophage content of different strains within M3-serotypes isolated from multiple geographical areas between 1920 and 2002 suggests that the successive gain of prophages over time has led to a contemporary M3 strain with increased virulence [14]. Additionally, multiple research groups have provided strong evidence to demonstrate that the rise of the hyper-virulent M1T1 clone in the late 1980s can be attributed, at least in part, to the horizontal acquisition of a 36-kb chromosomal segment (from a serotype M12 strain) and two separate bacteriophages, encoding different virulence determinants: a highly-active secreted DNase (Sda1/SdaD2) and the superantigen (SpeA) [10, 33–37].

These analyses highlight the importance of bacteriophages in streptococcal diversity, and provide preliminary evidence for a mechanism by which certain “virulent clones” could quickly evolve through horizontal transfer of prophage-like elements. Additionally, phage can play a more general role in evolution as a mechanism of horizontal gene transfer, through generalized transduction and recombination within the streptococcal chromosome [15, 36, 38, 39].

A comparison of isogenic strains of *S*. *pyogenes* that differ in specific bacteriophage genes or in overall bacteriophage content would allow us to study the direct effects that individual bacteriophage have on streptococcal virulence and may provide insight into the evolution of pathogenicity of specific strains in relation to prophage integration or loss.

In this study, we describe a genetic methodology that specifically manipulates the presence of integrated Group A streptococcal bacteriophage and SpyCIM1 elements, to produce isogenic mutants that are consecutively cured of prophages and differ only in prophage and prophage-like content. In total, we constructed 15 different knockout (KO) mutants of the M1 strain SF370, which represent every possible combination of phage and SpyCIM1 deletions, and additionally created the first mutant *S*. *pyogenes* strain (CEM1ΔΦ) that is completely cured of all major bacteriophage-like elements (a loss of ~10% of the original genome). We then compared the full phage KO (CEM1ΔΦ) to the wild type (SF370SmR) to examine a subset of contributions that could be attributed to prophage presence in the *S*. *pyogenes* genome, such as morphology, *in vitro* growth rate, secretion of phage encoded virulence factors, and lytic and lysogenic bacteriophage interactions.

## Materials and Methods

### Bacterial Strains and Growth Conditions

*S*. *pyogenes* strain SF370 (an M1 serotype) was isolated from a wound infection and kindly provided by J. Ferretti, University of Oklahoma Health Sciences Center [2]. A spontaneous streptomycin-resistant derivative of SF370 (discussed below) will be referred to as SF370SmR throughout these studies. All of the isogenic phage deleted mutants were derived from this *S*. *pyogenes* background (Table 1). One Shot® TOP10 *E*. *coli* (strain DH5α) (Invitrogen / ThermoFisher Scientific, Grand Island, NY) was used for plasmid construction and vector propagation.

**Table 1:** Strains described in this study.

Strain^a^	Bacteriophage and/or gene deleted^b^	Antibiotic resistance phenotype^b^	Refererence or source
***Streptococcus pyogenes***			
SF370	WT—None	Kan^S^ Sm^S^	[7]
SF370SmR	WT—None	Kan^S^ Sm^R^	This study
**WT + counter-selection cassette**			
CEM1KRΔ*speC*	*speC* (Φ370.1 superantigen ORF)	Kan^R^ Sm^S^	This study
CEM1KRΔ*speH*	*speH* (Φ370.2 superantigen ORF)	Kan^R^ Sm^S^	This study
CEM1KRΔ*spd3*	*spd3* (Φ370.1 DNase ORF)	Kan^R^ Sm^S^	This study
CEM1KRΔ*spy2136*	*spy2136* (SpyCIM1 primase ORF)	Kan^R^ Sm^S^	This study
**Single phage KOs**			
CEM1Δ1	Φ370.1	Kan^S^ Sm^R^	This study
CEM1Δ2	Φ370.2	Kan^S^ Sm^R^	This study
CEM1Δ3	Φ370.3	Kan^S^ Sm^R^	This study
CEM1Δ4	SpyCIM1	Kan^S^ Sm^R^	This study
**Single phage KOs + counter-selection cassette**			
CEM1KRΔ2-*speC*	Φ370.2, *speC*	Kan^R^ Sm^S^	This study
CEM1KRΔ1-*spd3*	Φ370.1, *spd3*	Kan^R^ Sm^S^	This study
CEM1KRΔ1-*spy2136*	Φ370.1, *spy2136*	Kan^R^ Sm^S^	This study
CEM1KRΔ2-*spd3*	Φ370.2, *spd3*	Kan^R^ Sm^S^	This study
CEM1KRΔ2-*spy2136*	Φ370.2, *spy2136*	Kan^R^ Sm^S^	This study
CEM1KRΔ4-*spd3*	SpyCIM1, spd3	Kan^R^ Sm^S^	This study
**Double phage KOs**			
CEM1Δ1,2	Φ370.1, Φ370.2	Kan^S^ Sm^R^	This study
CEM1Δ1,3	Φ370.1, Φ370.3	Kan^S^ Sm^R^	This study
CEM1Δ1,4	Φ370.1, SpyCIM1	Kan^S^ Sm^R^	This study
CEM1Δ2,3	Φ370.2, Φ370.3	Kan^S^ Sm^R^	This study
CEM1Δ2,4	Φ370.2, SpyCIM1	Kan^S^ Sm^R^	This study
CEM1Δ3,4	Φ370.3, SpyCIM1	Kan^S^ Sm^R^	This study
**Double phage KOs + counter-selection cassette**			
CEM1KRΔ1,2-*spd3*	Φ370.1, Φ370.2, *spd3*	Kan^R^ Sm^S^	This study
CEM1KRΔ1,2-*spy2136*	Φ370.1, Φ370.2, *spy2136*	Kan^R^ Sm^S^	This study
CEM1KRΔ1,4-*spd3*	Φ370.1, SpyCIM1, spd3	Kan^R^ Sm^S^	This study
CEM1KRΔ2,3-*spd3*	Φ370.2, SpyCIM1, spd3	Kan^R^ Sm^S^	This study
**Triple phage KOs**			
CEM1Δ1,2,3	Φ370.1, Φ370.2, Φ370.3	Kan^S^ Sm^R^	This study
CEM1Δ1,2,4	Φ370.1, Φ370.2, SpyCIM1	Kan^S^ Sm^R^	This study
CEM1Δ1,3,4	Φ370.1, Φ370.3, SpyCIM1	Kan^S^ Sm^R^	This study
CEM1Δ2,3,4	Φ370.2, Φ370.3, SpyCIM1	Kan^S^ Sm^R^	This study
**Triple phage KOs + counter-selection cassette**			
CEM1KRΔ1,2,4-*spd3*	Φ370.1, Φ370.2, SpyCIM1, *spd3*	Kan^R^ Sm^S^	This study
**Quadruple phage KO**			
CEM1ΔΦ	Φ370.1, Φ370.2, Φ370.3, SpyCIM1	Kan^S^ Sm^R^	This study
**Re-Lysogenized strains**			
CEM1Δ1−C1	Φ370.1 (+ Φ370.1)	Kan^S^ Sm^R^	This study
CEM1ΔΦ−C1	Φ370.1, Φ370.2, Φ370.3, SpyCIM1, (+ Φ370.1)	Kan^S^ Sm^R^	This study
***Escherichia coli***			
One Shot DH5α	Used for cloning and propagation of the counter-selection vectors		Invitrogen

^a^ Phage number or gene to the right of the Δ symbol have been deleted; KR, contains counter-selection cassette (*(aacA-aphD)* / *rpsL*^WT^).

^b^ Abbreviations used: WT, wildtype; ORF, open reading frame; (+ Φ370.1), gain of Φ370.1; Kan^R^, kanamycin-resistant; Kan^S^, kanamycin-sensitive; Sm^R^, streptomycin-resistant; Sm^S^, streptomycin-sensitive.

*E*. *coli* was cultured in Luria-Bertani (LB) broth and on LB agar at 37°C. *S*. *pyogenes* strains were grown at 37°C without shaking in Brain Heart Infusion (BHI) broth or Todd Hewitt Broth plus 2% yeast extract (THY) medium and on Proteose Peptone No.3 supplemented with 4% defibrinated sheep blood (Cleveland Scientific, Bath, OH) (PPB) agar or Columbia Blood Agar plates. When required, media was supplemented with antibiotics at the following concentrations: erythromycin at 200 μg/ml for *E*. *coli* and 15 μg/ml for *S*. *pyogenes*; kanamycin at 50 μg/ml for *E*. *coli* and 250 μg/ml for *S*. *pyogenes*; streptomycin at 200 μg/ml for *S*. *pyogenes*. All antibiotics were supplied by Sigma-Aldrich (St. Louis, MO). All media was supplied by Difco (BD, Becton, Dickinson and Company, Hunt Valley, MD) unless stated otherwise.

### DNA Manipulations

Streptococcal genomic DNA was isolated with either the DNeasy Tissue Kit or the Blood & Cell Culture DNA Kit (Qiagen, Germantown, MD) following the manufacturer’s protocols, except for the substitution of a modified lysis buffer (50 mM Tris-Cl pH 6.6, 50 mM EDTA, 0.5% Tween-20, 0.5% TritonX-100) supplemented with 500U of PlyC, a streptococcal bacteriophage lysin [40] and 250 ng/ml of RNase A (Qiagen). Plasmid DNA was isolated from *E*.*coli* using the QIAprep Spin Miniprep Kit or HiSpeed Plasmid Midi Kit (Qiagen). DNA fragments were gel purified from 1% Agarose gels using the QIAquick Gel Extraction Kit (Qiagen). T4 DNA ligase and all restriction enzymes, unless otherwise stated, were purchased from New England Biolabs (Ipswich, MA) and used according to the manufacturer’s instructions unless otherwise stated. Oligonucleotide primers were obtained from Fisher Scientific-Operon (Pittsburgh, PA) and are listed in (S1 Table). PCR was performed using AmpliTaq Gold DNA polymerase, Gold Buffer, 1.5 mM MgCl2, and 200 μM dNTPS (Applied Biosystems, Foster City CA) following standard protocols with the Eppendorf Mastercycler (Eppendorf, Hauppauge, NY). Southern blot analysis utilized probes from the primers & PCR products described in (S1 Table) with the Amersham ECL Direct Nucleic Acid Labeling and Detection System (GE Healthcare Biosciences, Pittsburgh, PA). DNA sequencing was performed by GENEWIZ, Inc. (North Brunswick, NJ). DNA sequence analysis, comparison, and manipulation were accomplished with Lasergene software modules (DNASTAR Inc., Madison, WI). DNA primers were designed with MacVector software (Accelrys Inc., San Diego, CA).

### Isolation of SF370 with a Genomic Mutation Conferring Streptomycin Resistance

A spontaneous streptomycin-resistant mutant of strain SF370 was selected by serial passage in BHI containing increasing concentrations of the antibiotic (0–200 μg/ml), followed by growth on PPB agar containing 200 μg/ml streptomycin. In order to confirm that a mutation in the *rpsL* gene caused the Sm^R^ phenotype, the gene was PCR amplified and the DNA sequence compared to published *rpsL* sequences from the SF370 genome and other types of Sm^R^ bacteria, [41–44]. The resulting Sm^R^ strain was used in all subsequent bacteriophage deletion experiments and is referred to as SF370SmR.

### Construction of Bacteriophage Counter-Selection Vector, pFWKR

To generate the counter-selection vector, the 676 bp DNA sequence of the SF370 *rpsL*^WT^ gene and upstream promoter region, was inserted between the kanamycin resistance gene *(aacA-aphD)* and a downstream multiple cloning site (MCSII) of the streptococcal shuttle vector pFW13 [45], as described in the (S1 Fig). The recombinant vector was transformed into *E*. *coli* and transformants selected on LB agar containing kanamycin. Plasmid DNA was isolated from the Kan^R^ cultures and sequenced to verify proper insertion of the WT *rpsL* gene into the vector. The resulting two-gene (*(aacA-aphD)* / *rpsL*^WT^) positive/negative selection cassette was designated as a “Janus cassette” based on a similar counter-selection cassette used for gene replacement in *Streptococcus pneumoniae* [46]. The plasmid, which contained our Janus cassette, was named pFWKR and served as the backbone to construct all other phage counter-selection vectors (S1 Fig).

### Counter-Selection Step 1: Allelic Replacement of a Bacteriophage ORF with the pFWKR Janus Cassette

The *speH* superantigen gene encoded by phage Φ370.2 was chosen first for allelic replacement since we previously showed it could be successfully replaced with other antibiotic resistance genes [47]. Following the allelic replacement protocols described above and published previously [47] the *speH* gene was replaced with the Janus cassette contained in the pFWKR vector above. To accomplish this, the DNA regions flanking *speH* were separately PCR amplified from SF370 (662 bp upstream and 888 bp downstream) using the two primer sets described by Ryan *et al*. (S1 Table) [47]. The amplicons and pFWKR vector were digested with the corresponding restriction enzymes, gel purified, and the individual upstream and downstream fragments were cloned into the MCSI and MCSII, respectively. The resulting plasmid DNA was PCR amplified and sequenced to confirm the creation of this pFWKR-speH vector (S1 Table). The pFWKR-speH vector was then electroporated into strain SF370 following the protocol of Kimoto and Taketo [48], and transformants were selected on PPB containing kanamycin. Kan^R^ colonies were then plated on PPB agar containing kanamycin (250 μg/ml) and streptomycin (200 μg/ml) to verify Sm^S^, conferred by the WT *rpsL* gene in the replacement cassette. Double mutants (Kan^R^/Sm^S^) were screened by PCR and Southern blot analyses to verify the proper allelic replacement of the *speH* gene with the Janus cassette (data not shown). The resulting mutant, CEM1KRΔspeH, was then used to screen for the loss of the entire Φ370.2 bacteriophage (Table 1).

### Counter-Selection Step 2: Selection of Mutants Cured of Bacteriophage

To cure the CEM1KRΔspeH mutant of the Φ370.2 phage, a single colony was inoculated into BHI broth, without antibiotics, and grown overnight at 37°C. The overnight culture was plated onto PPB agar containing streptomycin (200 μg/ml), and once again incubated overnight at 37°C to select for Sm^R^ colonies that potentially lost the phage harboring the Janus cassette. To confirm cassette loss and eliminate false positive Sm^R^ colonies, caused by point mutations or gene conversion events in the *rpsL*^WT^ gene, Sm^R^ colonies were replica plated to PPB agar containing kanamycin to identify isolates that were also Kan^S^. The (Sm^R^ / Kan^S^) mutants were then verified for complete loss of the Φ370.2 phage by PCR (using primer sets spanning both sides of the bacteriophage integration or attachment sites) (S1 Table), Southern blot hybridization analysis (with Φ370.2 specific gene probes), DNA sequence analysis, and PFGE analysis. The resulting mutant strain, CEM1Δ2, no longer contained the integrated Φ370.2 DNA in the streptococcal chromosome (Table 1).

### Selection for the Loss of the Three Additional Bacteriophage-Like Elements from SF370

Unless otherwise stated in the results, the two-step method to cure SF370SmR of the three remaining prophage was accomplished using the same techniques described above for the deletion of the Φ370.2 bacteriophage, except that the counter-selection pFWKR-like plasmids for each remaining prophage element were constructed using a combination of PCR based fusion assembly and cloning methods adapted from S. Lizano *et al*. [49, 50]. Briefly, instead of restriction digestion and individual ligation into the MCSs of pFWKR, the corresponding amplicons of the upstream and downstream DNA regions (500 bp– 1400 bp) adjacent to each of the virulence genes from the individual prophage were fused to both sides of a third PCR amplicon that contained the Janus cassette from plasmid pFWKR. Full-length products were then amplified by another round of PCR with nested primers that had homology to the far ends of the adjacent upstream and downstream regions and contained a NheI or XmaI site. The fused amplicons, containing the Janus cassette flanked by phage DNA, and the pFWKR plasmid were then double-digested with the corresponding enzymes and ligated together to derive the counter-selection plasmids similar in composition to the pFWKR-speH vector above (Plasmids and Primers summarized in S1 Table). The resulting vectors were used for transformation and allelic replacement in SF370SmR, CEM1Δ2, and each subsequent bacteriophage deletion mutant. In each case, our Janus cassette was individually inserted into the remaining prophage, replacing virulence genes, (*speC*, Φ370.1; *spd3*, Φ370.3,) or the replicase gene (*spy2136*) of SpyCIM1. After growth and antibiotic counter-selection, proper phage loss in the Sm^R^/Kan^S^ colonies was confirmed by the above analyses (S1 Table and S2 Fig). For the derivation of the multiple phage deletion mutants, the experimental progression of phage counter-selection and consecutive phage loss always followed the order of: Φ370.2, Φ370.1, SpyCIM1, and then Φ370.3 (first to last respectively). This process was repeated until every permutation of phage loss was obtained (15 mutants in all), including a strain that was devoid of all major bacteriophage-like DNA sequences named CEM1ΔΦ (summarized in Table 1).

### PFGE Molecular Analysis

Agarose discs of genomic DNA from SF370SmR and mutant isolates (Table 1) were prepared according to a protocol modified from Chung *et al*. [51], in which 500U of PlyC was substituted for lysozyme and lysostaphin enzymes in the cell lysis solution [40]. DNA was digested with SmaI overnight at 25°C and subjected to PFGE with the CHEF-DR II system (Bio-Rad, Hercules CA) as previously described [51, 52]. DNA bands in the agarose gel were visualized with ethidium bromide and photographed with an Alpha imager (Alpha Innotech Corp., San Leandro, CA). DNA banding patterns were analyzed by previously described methods [51, 53].

### Comparison of DNase Activity in Bacteriophage Cured Strains

Overnight cultures of SF370SmR and CEM1ΔΦ grown in BHI or individual colonies grown on Columbia Blood Agar plates were streaked for isolation onto DNase Test Agar with Methyl Green (Difco). After overnight incubation at 37°C, the size of clearing zones surrounding colonies, which indicated DNase activity on the DNA-methyl green substrate in the agar, were compared for a qualitative assessment of DNase activity of each strain.

### Mitomycin C Induction of Prophage

Prophage were induced using a modification of the method of McShan *et al*. [54]. Briefly, WT SF370 (Sm^S^), CEM1ΔΦ, and other phage cured mutants were grown overnight at 37°C in BHI broth. The overnight culture was diluted 1:30 (v/v) in 100 ml of BHI broth and incubated at 37°C to an O.D._600_ of 0.2. The culture was then divided, one half received mitomycin C (Sigma) at 0.2 μg/ml and the other half served as a control. After two hours at 37°C the optical density of the mitomycin C induced culture of SF370 began to drop. This decrease was not observed in either the untreated controls or the mitomycin C treated CEM1ΔΦ cultures (data not shown). Cells were then pelleted by centrifugation and supernatants collected and sterile-filtered through 0.22 μm filters. Filtrates were concentrated and buffer exchanged with 100,000 MWCO Amicon Ultra-15 centrifugal filter devices (Millipore) to achieve a final volume of 500 μl in prophage suspension buffer [55] (0.15 M NaCl, 10 mM Tris HCl [pH 7.5], 5 mM MgCl2, 1 mM CaCl2) These concentrated phage stocks were stored at 4°C for a maximum of 72 hours before being used.

### Analysis of Bacteriophage Plaques and Lysogens

Potential indicator strains, (SF370SmR, CEM1ΔΦ, and the single phage KO mutants in Table 1) were grown overnight at 37°C in BHI broth containing 200 μg/ml of streptomycin. Aliquots (100 ul) of these cultures were added to 3 ml of melted BHI soft agar (7.5%) at 50°C, mixed, and poured on top of PPB agar plates. After the agar solidified, 10 μl of the concentrated phage stocks were spotted onto the plates. Phage supernatants spotted to PPB agar plates alone (without indicator strain) served as control for bacterial contamination. Plates were incubated overnight (37°C plus 5% CO_2_) and analyzed for growth inhibition and bacteriophage plaques The same extent of growth inhibition or killing was not observed in concentrated control media containing only mitomycin C.

To screen for lysogens, colonies around the edge of the plaque clearing zones, or non-plaque areas where lysates were dropped on lawns, were picked and streaked for isolation on PPB agar plates containing streptomycin (200 μg), to inhibit WT SF370 (Sm^S^) bacterial contamination. Streptomycin resistant colonies were then analyzed for lysogeny by PCR amplification, using primers specific to bacteriophage genes from each of the four major elements of SF370 (S1 Table). Colonies that had positive amplicons were further analyzed for proper phage integration using PCR primers specific for the SF370 phage attachment sites (S1 Table). The genomic DNA from putative lysogens was then subjected to PFGE analysis to ensure there were no other non-specific integration events or gross chromosomal arrangements.

## Results

### Principle of Prophage Counter-Selection Technique

To isolate mutants cured of prophage, we used allelic replacement techniques to exchange individual prophage genes with a two-gene Janus cassette containing: 1) the *(aacA-aphD)* gene, encoding kanamycin resistance (Kan^R^) [45] and 2) the wild-type *rpsL* gene (*rpsL*^WT^), encoding the ribosomal subunit protein S12, the target of the antibiotic streptomycin [46, 56]. While point mutations in the chromosomal *rpsL* gene provide a high level of resistance to streptomycin (Sm^R^), the resistance is recessive if a second copy of the gene (*rpsL*^WT^) is also expressed in the same cell [56, 57]. Therefore, if the *rpsL*^WT^ gene is introduced by allelic replacement into a prophage of a strain harboring a chromosomal *rpsL* mutation (naturally Sm^R^), the strain will become streptomycin sensitive (Sm^S^). Such a system makes it possible to select for rare clones that spontaneously lose the prophage-inserted copy of the *rpsL*^WT^ allele and to counter-select against genetic elements that harbor a copy of *rpsL*^WT^ by plating the bacteria on media containing streptomycin [56]. Isolates that retain the *rpsL*^WT^ copy (i.e. the prophage) will be Sm^S^, while strains that are spontaneously cured of the prophage carrying the *rpsL*^WT^ gene will be Sm^R^.

### Selection for the Loss of Bacteriophage and SpyCIM1

We first isolated a mutant strain of SF370 that had become spontaneously resistant to streptomycin as described in the Methods section. DNA sequence analysis of the *rpsL* region of this mutant revealed a single point mutation in the *rpsL* gene (lysine 56 to arginine (K56R)), which is consistent with *rpsL* mutations that confer streptomycin resistance in other bacterial species [42–44, 46]. No gross differences were observed in growth rate or morphology between the streptomycin resistant mutant (SF370SmR) and WT strain of SF370 (data not shown).

**Fig 2:**
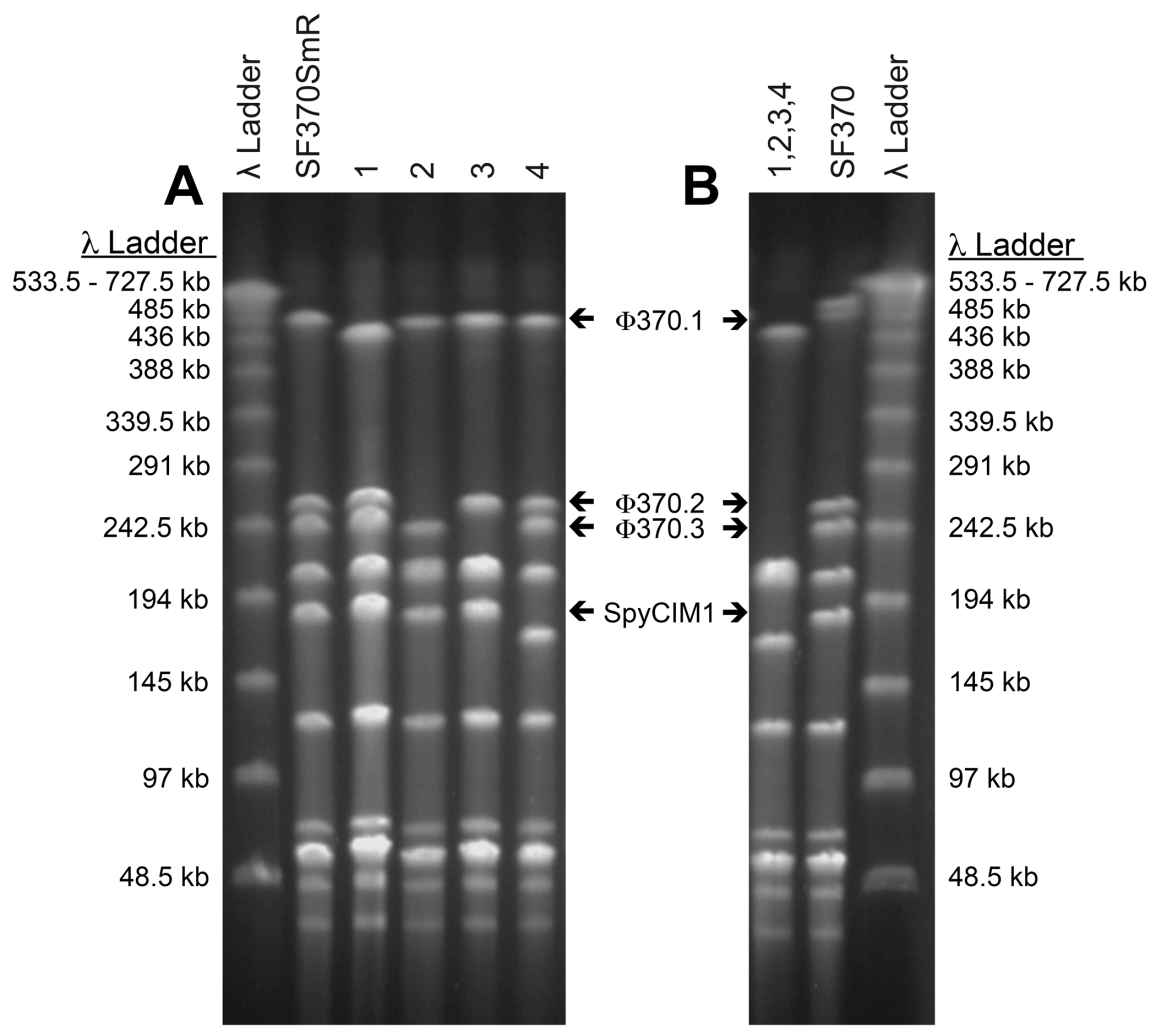
Pulse field gel electrophoresis (PFGE) analysis of *S*. *pyogenes* SF370SmR and single phage deletion mutants. PFGE patterns of SmaI digested genomic DNA from A, the phage wild type (WT) strain SF370SmR and the single phage KOs CEM1Δ(1–4), and B, the quadruple full phage KO CEM1ΔΦ compared to WT strain SF370. Labeled arrows indicate DNA fragments that contain the corresponding integrated phage or phage-like elements, based on the genome sequence of SF370. A loss or drop of these DNA fragments represents a phage or SpyCIM1 deletion. Numbers at top indicate which phages have been deleted: 1, Φ370.1 (40.9kb), strain CEM1Δ1; 2, Φ370.2 (42.5 kb), strain CEM1Δ2; 3, Φ370.3 (33.5Kb), strain CEM1Δ3; 4, Φ370.4/SpyCIM1 (13.5kb), strain CEM1Δ4; (1,2,3,4), all 4 phage elements deleted to create strain CEM1ΔΦ. All lanes in A and B originated from the same PFGE gel as shown in (S2 Fig). λ, Lambda ladder, New England Biolabs PFG Marker with DNA fragment sizes on adjacent to gel images.

Next, SF370SmR was transformed with the counter-selection vector, pFWKR-speH (S1 Table), to replace the *speH* gene in Φ370.2 with the Janus cassette and create the Kan^R^/Sm^S^ mutant CEM1KRΔ*speH* (Table 1). This mutant was grown overnight in media without antibiotics and plated on streptomycin agar plates to select for an isolate that spontaneously lost the Φ370.2 prophage, containing the Janus cassette, thus reverting back to the Kan^S^/Sm^R^ phenotype of SF370SmR. Complete loss of the Φ370.2 phage DNA and absence of gross chromosomal alterations were confirmed for this mutant (and all subsequent mutants) by four different methods: 1) lack of PCR amplification products specific for Φ370.2 genes (S1 Table), 2) Southern blot analysis with Φ370.2 phage specific probes described in S1 Table (data not shown), 3) PFGE analysis (Fig 2), and 4) sequencing across the *attB* phage integration site.

The resulting mutant strain, CEM1Δ2 (Table 1), lacked the integrated Φ370.2 DNA and recapitulated the predicted *attB* bacterial attachment site from the SF370 WT genome sequence (Table 2). Furthermore, the nucleotide sequence of this region was identical to that of a modern M1 strain, MGAS5005, which does not contain a bacteriophage integrated into the homologous genome location [2, 10].

**Table 2:** Confirmed phage *attB* sequences in *S*. *pyogenes* SF370 prophage KO mutants.

Prophage	Prophage-KO *attB* sequences^a^
Φ370.1	CATGTACAACTATACT
Φ370.2	AACTCAAGAAGTGATTAAATAAAACATTAAAGAACCTTGTCATATCAACG
Φ370.3	AATTATTTAACAGCGTCTTT
SpyCIM1	CAATAATGTTTGTCATAATTT

^a^ All sequences are listed 5' to 3' on the forward strand of streptococcal DNA sequences and match predicted *attB* from SF370, as well as *attB* from other published *S*. *pyogenes* genomes that lack prophage at these sites. *attB*, phage attachment sites on bacterial chromosome.

To select for loss of the Φ370.1 phage, the *speC* gene was replaced with the Janus cassette to create the mutant CEM1Δ*speC*. This strain was then passaged and subjected to our counter selection methods to isolate colonies that had a potential phage deletion. Genomic DNA from these clones was screened by the four confirmatory methods listed above to verify that Φ370.1 was lost and that there were no aberrant recombination events in the production of the mutant named CEM1Δ1 (Fig 2 and Tables 1 and 2). Using this same procedure, CEM1Δ2 was also cured of Φ370.1 to produce the double phage (Φ370.1 and Φ370.2) deletion mutant CEM1Δ1,2. (Table 1 and S2 Fig).

To select for loss of SpyCIM1 we replaced the phage primase gene (*spy2136*), with the Janus cassette to create the CEM1KRΔSpy2136 mutant (Table 1). We hypothesized that deletion of the primase gene might interfere with replication of the excised circular form of SpyCIM1, potentially promoting loss of the element during cell division. However, all resulting mutants (> 500 CFU) were Sm^R^/ Kan^R^, suggesting a rate of recombination, gene conversion or mutation in the *rpsL*^WT^ gene of the Janus cassette was significantly higher than the rate of loss of SpyCIM1 [46]. This confirmed previous results that SpyCIM1 was difficult to cure from SF370 [5, 7, 58].

To increase the probability of isolating a mutant cured of SpyCIM1 we adapted a plasmid curing protocol from [59], in which CEM1KRΔSpy2136 cultures were. incubated in BHI at 42°C for 17 hours, then subsequently plated to streptomycin PP3 agar at 37°C. Resulting Sm^R^/ Kan^S^ colonies were confirmed to have lost SpyCIM1 by PCR, Southern blot and PFGE analyses (data not shown). One such mutant was chosen for further analysis and was designated CEM1Δ4 (Table 1 and Fig 2). Of note, compared to the wild type background, SpyCIM1 was lost more efficiently from the other prophage-deleted mutants, CEM1Δ1, CEM1Δ2, and CEM1Δ1,2 using our standard counter-selection protocol at 37°C. This suggests that the other prophage may act as helper phages that aid in the maintenance and/or spread of SpyCIM1. The resulting double and triple phage cured mutants are listed in (Table 1) and PFGE patterns represented in (S2 Fig).

To isolate a deletion mutant of Φ370.3 the *spd3* gene was replaced with the Janus cassette to create the CEM1KRΔ*spd3* mutant, followed by counter selection to obtain strain CEM1Δ3 (Table 1), cured of Φ370.3 (Fig 2 and Table 2). This phage was also deleted from the preceding single and double mutants using the same vector and methods (Table 1). Finally, the triple KO mutant (CEM1Δ1,2,4), was similarly manipulated to select for the loss of Φ370.3 and derive the first Group A streptococcal strain devoid of all phage and phage-like elements, named CEM1ΔΦ (Table 1, Fig 2 and S2 Fig).

### Phenotypic Comparison and Decreased Production of DNase in Bacteriophage-Free Mutant CEM1ΔΦ

In our first set of analyses, we examined the basic phenotypes of CEM1ΔΦ as compared to the SF370SmR wild type strain. We found no significant differences in microscopic appearance of cells including morphology or streptococcal chain length, or in macroscopic characteristics such as colony morphology or hemolytic patterns on sheep blood agar. Furthermore, the growth curves produced during liquid culture in enriched laboratory media (THY and BHI) did not reveal appreciable differences in growth rate (data not shown).

**Fig 3:**
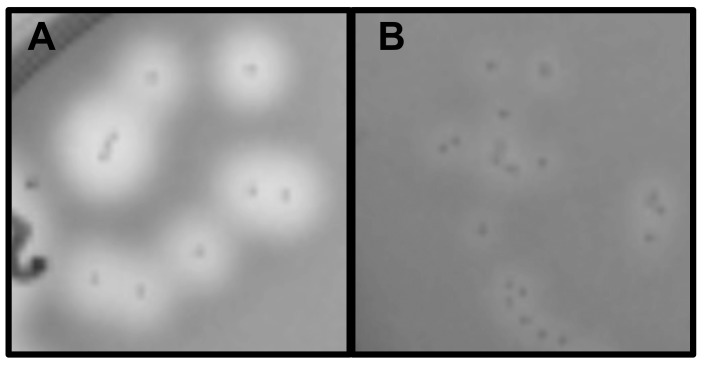
Secreted DNase activity of SF370SmR and CEM1ΔΦ. Both strains were cultured on the same DNase Test Agar plate with Methyl Green for 17 h. A, SF370SmR WT colonies; B, CEM1ΔΦ full phage KO. Clearing zones around the colonies resulted from hydrolysis of the DNA-methyl green substrate, and signify DNase activity.

Next, we assessed the production of virulence factors whose expression might be modulated by the lack of prophage. As one example, we examined the secretion of streptococcal DNases, which have been shown to affect survival, virulence, and pathogenicity in other *S*. *pyogenes* strains [35, 60]. The genome of SF370 (SF370SmR) encodes three secreted DNases: one that is chromosomally encoded (*spd/mf1*) and two others that are phage-encoded (*spd1* on Φ370.1 and *spd3* on Φ370.3) [2, 3, 61]. To assess the production of the phage-encoded DNases as compared to the chromosomal encoded gene alone, strain CEM1ΔΦ and the wild-type strain SF370SmR were plated on DNase Test Agar with Methyl Green. After overnight growth, colonies from SF370SmR produced larger and more distinct clearing zones of DNA degradation than the CEM1ΔΦ colonies (Fig 3 and S3 Fig). This suggests that the phage DNases contribute to the total secreted DNase activity of the wild-type strain and/or the presence of prophage may influence the expression and/or secretion of the chromosomally encoded DNase.

### Interactions of CEM1ΔΦ with SF370 Induced Bacteriophage

To test if the CEM1ΔΦ phage-cured mutant could be re-infected, lysed and/or lysogenized by induced SF370 WT bacteriophages, concentrated phage supernatants from mitomycin C induced or non-induced cultures were tested on SF370SmR or CEM1ΔΦ indicator strains. When applied to the SF370SmR indicator strain, there was no substantial growth inhibition or plaque formation observed for any of the six phage supernatants listed in Table 3. Likewise, none of the supernatants from any non-induced cultures or from the mitomycin C induced CEM1ΔΦ showed discernable growth inhibition or plaque formation on the CEM1ΔΦ indicator. However, the supernatant from mitomycin C treated SF370 WT strain did produce significant bacterial clearance and/or plaque formation on the lawn of the CEM1ΔΦ indicator strain (Table 3).

**Table 3:** Lytic potential of phage KO mutants.

Mitomycin C Induced Φ Lysate	Indicator Lawns^a^					
	SF370SmR	CEM1ΔΦ	CEM1Δ1	CEM1Δ2	CEM1Δ3	CEM1Δ4
**SF370**	**-**	**+**	**+**	**-**	**-**	**-**
**CEM1ΔΦ**	**-**	**-**	**-**	**-**	**-**	**-**
**CEM1Δ2,3,4**	**-**	**+**	**+**	**N/A**	**N/A**	**N/A**
**CEM1Δ1,2,3**	**-**	**-**	**-**	**N/A**	**N/A**	**N/A**
**CEM1Δ1,2,4**	**-**	**-**	**-**	**N/A**	**N/A**	**N/A**
**CEM1Δ1,3,4**	**-**	**-**	**-**	**N/A**	**N/A**	**N/A**

^a^ +, positive plaque clearing zones; -, no substantial plaque clearing zones; N/A, not performed

To assess which SF370 bacteriophage was involved in plaque formation on CEM1ΔΦ, concentrated supernatants from mitomycin C-induced SF370 cultures were dropped onto soft agar overlays that contained SF370SmR, each of the single phage-deleted mutants (CEM1Δ1, CEM1Δ2, CEM1Δ3, and CEM1Δ4), or CEM1ΔΦ. Substantial plaque clearing zones were only observed on the lawns of the CEM1ΔΦ and CEM1Δ1 mutants (Table 3). Since plaque formation is often inhibited by superinfection exclusion [62], these results suggested that the Φ370.1 phage was responsible for the majority of plaque clearing zones on the lawns of CEM1ΔΦ. This interpretation was further supported by the formation of plaques on CEM1ΔΦ using mitomycin C induced lysate from strain CEM1Δ2,3,4, which only contains the Φ370.1 phage. No plaques or clearing zones were observed on CEM1ΔΦ with any of the other triple phage KO mutants that contained only one of the other prophages-like elements (Φ370.2, Φ370.3, or SpyCIM1) (Table 3).

To assay for lysogeny, CEM1ΔΦ or CEM1Δ1 colonies were picked and plated for isolation from the plaque borders of lawns treated with the Mitomycin C induced phage lysate of SF370. Additionally, lawn regions of the other single phage KO mutants that were treated with SF370 phage lysate, but were negative for plaque formation were also picked and plated for isolation. Approximately 100 colonies (Sm^R^) of each recipient indicator strain were screened for integrated phage by PCR analysis with primers specific for each of the four elements. While we did not detect any Φ370.2 or Φ370.3 bacteriophage genes, we identified the Φ370.1 phage *speC* gene in DNA from the colonies of CEM1ΔΦ and CEM1Δ1. From these isolates, lysogeny and proper phage integration into the original Φ370.1 (*attB*) site were confirmed by PCR, DNA sequence analysis, and PFGE (data not shown). Two lysogens, which retained the integrated Φ370.1 prophage in the chromosome after successive passage, were designated CEM1ΔΦ-C1 and CEM1Δ1-C1 (Table 1). Additionally, during the course of these studies, further PCR screens of potential CEM1ΔΦ lysogens also identified the *spy2136* primase gene of SpyCIM1. These preliminary results indicate that this element might also be transferred and possibly integrate into the genome of the CEM1ΔΦ mutant (data not shown). In-depth analyses of these findings are currently underway.

## Discussion

Comparative genomic studies of the published sequences of *S*. *pyogenes* isolated from diverse streptococcal infections have confirmed the important relationship between *S*. *pyogenes* and its lysogenic bacteriophages [20, 24, 33, 34, 55]. While phage are known to play a role in streptococcal pathogenesis because of their encoded virulence factors, the overall molecular and genetic interactions between phage and the host have not been adequately explored [35, 60, 63–66]. As such, a comparison of isogenic strains of *S*. *pyogenes* that differ in specific bacteriophage content would allow us to study the direct effects that individual bacteriophage have on the streptococcal genome and pathogenesis.

Previous methods promoting phage loss in other bacterial species have relied on the use of DNA-damaging reagents to activate the bacterial SOS response (i.e., mitomycin-C, ethidium bromide or UV light), which increase both phage induction and the rate of loss of the integrated phage from the genome [67–69]. However, because the nature of these techniques induces DNA damage, they have the potential to cause mutations in other areas of the bacterial chromosome, leading to false phenotypes or misleading conclusions regarding the differences observed between two potential isogenic mutants. These methods also fail to give the researcher control over which phage are cured from poly-lysogenized bacteria, including *S*. *pyogenes*. While simply passaging and screening large numbers of bacterial colonies for phage loss by PCR may be useful for other species [70], we, along with others in the Group A streptococcal field, have found such non-selective methods to be unacceptable for isolating *S*. *pyogenes* mutants that differ in lysogeny (unpublished results).

The counter-selection technique presented here allows for precise manipulation of lysogeny by selecting for spontaneous loss of targeted phage in a way that naturally recapitulates or preserves the *attB* of each prophage integration site. Additionally, by targeting potential virulence factors or other phage genes of interest for allelic replacement with the Janus cassette, with relatively little extra effort the investigator can also use the primary Kan^R^/Sm^S^ isolates to interrogate an individual gene KO, before or after a phenotype is seen in a phage cured strain. Thus, from the same cloned KO vector and one streptococcal transformation (allelic replacement) procedure, it is also possible to screen phenotypes associated with the loss of a single phage gene or the whole bacteriophage genome.

Furthermore, because the loss of phage is concomitant with the loss of the antibiotic selection cassette (resulting in a marker-less phenotype), this method can be used to consecutively select for the loss of multiple prophage from the same bacterial chromosome. Previous experiments have taken advantage of this trait by inserting the *rpsL*^WT^ gene into plasmids for counter-selection against extra-chromosomal shuttle vectors and single crossover integration events in *S*. *pyogenes* [71]. Likewise, a similar counter-selectable cassette with a *rpsL*^WT^ gene and kanamycin resistance marker, originally termed a “Janus Cassette”, has been used to create silent (marker-less) mutations and gene deletions through gene replacement in *Streptococcus pneumoniae* [46]. While other comparable counter-selection or temperature-sensitive genes and methods have been applied to delete pathogenicity islands and bacteriophages from other genera of Gram-positive and Gram-negative bacteria [72–74], to our knowledge these types of methods have not been applied to delete specific prophage in Group A streptococci.

As a proof of concept, we created mutants of the *S*. *pyogenes* strain SF370SmR that varied in phage content as well as a mutant that was devoid of all of the major integrated bacteriophage elements (a loss of ~10% of the genome). In total we isolated 15 different mutants that comprise every permutation of the SF370SmR phage knockout (KO): single, double, and triple knockouts, in addition to the strain that was completely cured of all bacteriophage elements. To our knowledge, this is the first time such mutants of *S*. *pyogenes* have been successfully created, and serves as a novel method for creating isogenic mutants of *S*. *pyogenes* (or potentially other bacteria) that differ solely in phage content. Such KO strains will be useful tools to study the dynamics of phage lysogeny, the role phage play in both bacterial virulence and the regulation of chromosomal genes [58], and can provide insight into the evolution of streptococcal pathogenicity.

In the work presented here, we have demonstrated some preliminary examples of the diverse applications of these phage-cured mutants for analyzing the role of phage in *S*. *pyogenes* pathogenesis and to further study the molecular interactions between phage-encoded factors of streptococci. While it was shown for some species of bacteria and/or phage that the lysogenic state may affect growth or gross bacterial morphology [75, 76], our initial characterizations comparing the WT SF370SmR with the CEM1ΔΦ full phage KO did not detect differences in cell shape, chain length, colony morphology or growth rates *in vitro* using enriched media. Further analyses of the comparative growth of these isolates, in conditions that more closely replicate the *in vivo* environment, such as human saliva or blood, may yield different results. Moreover, differences may also be seen between the two strains in chemically defined media varying in metabolic constituents. These future experiments are supported by our ongoing transcriptional analyses comparing expression patterns of WT SF370SmR to the full phage KO (CEM1ΔΦ) and SpyCIM1 (CEM1Δ4) mutants. These studies indicate that presence of phage and phage-like elements affect many virulence and metabolic pathways encoded throughout the genome of *S*. *pyogenes* (data not shown) and [58].

### Virulence and DNase

The phage deletion mutants derived by our methods could be used to analyze the effects that the individual virulence factor genes encoded by prophage have on the survival of the host bacterium. As an example, we chose to compare the ability of wild type SF370SmR and CEM1ΔΦ to produce secreted DNases. DNases are thought to facilitate the spread of bacteriophage and bacteria [61, 77], degrade Neutrophil Extracellular Traps (NETs) [78] [60], and interfere with toll-like receptor pathways (i.e., TLR9) [79], to allow bacteria to escape and evade the host immune response [35]. Moreover, many groups have proposed that phage DNases (Sda1/2) play an important role in the survival and spread of the more pathogenic and contemporary M1 strains of *S*. *pyogenes* [10, 33–37]. As such, an analysis of the secreted DNases of SF370 may elucidate how the bacteriophage encoded DNases affect the virulence of this strain.

SF370 (SF370SmR) encodes three secreted DNases: the chromosomal DNase (*sdaB*/*spd*/*mf*) and two phage encoded enzymes, *spd1* from Φ370.1 and *spd3* from Φ370.3 [2, 61]. We compared DNase activity between the CEM1ΔΦ full phage KO, which lacks the *spd1* and *spd3* genes, and SF370SmR, which provided an *in vitro* qualitative assessment of DNase enzymatic activity. The analysis determined that the wild type SF370 is capable of degrading more DNA in vitro than CEM1ΔΦ, presumably due to a lack of phage DNase expression. Whether this effect is the direct result of a lack of phage encoded DNases and/or more complex interactions between phage, the chromosomal DNase gene, and bacterial regulators are questions currently being addressed. However, our preliminary transcriptional analyses have revealed no significant difference in the expression of the chromosomal encoded DNase (SdaB/Spd/MF) that could be attributed to the presence of prophage (data not shown). As such, a loss of DNase activity in the mutant seems to be directly associated with the loss of the two prophage-encoded DNases in CEM1ΔΦ. Further analysis of these phage deletion mutants are underway to study the regulation of other phage encoded virulence factors in both *in vitro* and *in* vivo models.

### Bacteriophage Lysis and Lysogeny of *S*. *pyogenes*

Isogenic phage-cured mutants could also be instrumental in studying numerous mechanisms of the phage life cycle (e.g., lytic and lysogenic infection), bacterial host defenses to viruses (e.g., Clustered regularly interspaced short palindromic repeat (CRISPR), Abortive infection (Abi) and Restriction-modification (RM) systems), phage-phage interactions (e.g., helper-viruses or super-infection exclusion), or other phenotypes associated with lysogenic phages that have inserted into the CEM1ΔΦ versus SF370SmR genome.

While SF370SmR contains two CRISPR-Cas loci, which provide resistance to infection from bacteriophage that have homologous DNA sequences to the CRISPR spacer sequences [80], we hypothesized that our phage-free mutant strain would be more susceptible to infection by other bacteriophage (which do not contain CRISPR homologies) since there are no prophage at the potentially shared *attB* integration sites [13, 32] or other phage-encoded proteins that might interfere with subsequent infections (e.g., bacteriophage CI-repressors and superinfection exclusion genes) [81]. To test this hypothesis, we exposed the mutant strains to supernatants from cultures of SF370 induced with mitomycin C and found that the phage cured CEM1ΔΦ mutant was capable of being re-infected and subsequently lysed by lytic bacteriophage. To further identify which phage was responsible for the lytic effect, SF370 phage supernatants where dropped onto different bacterial lawns of mutants lacking only one the four phage-elements. While other phage inducing agents may produce different results [82–84], plaque formation was only seen on the CEM1Δ1 mutant indicator strain, suggesting that Φ370.1 potentially was the only viable phage induced with mitomycin C. These results were supported by similar experiments that used induced supernatants from the four mutants with triple phage deletions, which each contained only one of the four phage-elements. Plaque formation was only seen in supernatants induced from the CEM1Δ2,3,4 mutant that contained only Φ370.1. Finally, we also isolated lysogens from the CEM1ΔΦ and CEM1Δ1 mutants that contained Φ370.1 re-integrated into the original a*ttB* site on the chromosome, proving the re-infection event.

These findings are in agreement with previous genetic analyses and experiments that suggest Φ370.1 is inducible after mitomycin C treatment [2, 3], while additionally proving that Φ370.1 was a fully functional bacteriophage capable of lytic infection and lysogeny. These results also proved that our phage-cured mutants, derived from our novel counter-selection method, were capable of being re-infected and/or lysogenized.

Furthermore, when screening CEM1ΔΦ for lysogeny, we also identified isolates that contained the DNA from SpyCIM1, suggesting that this element may be capable of being transferred by phage transduction. While the exact mechanism is unknown in *S*. *pyogenes*, elements like SpyCIM1 are often transferred by helper phage in other bacterial genera [8]. Interestingly, we found that it was much easier to obtain SpyCIM1 deletion mutants from our isolates in which the Φ370.1 and/or Φ370.2 prophage were previously lost from the genome, which supports a possible helper phage hypothesis.

Taken together, the results and methodology presented here illustrate the utility of these mutants in analyzing interactions between different streptococcal bacteriophage and/or other chromosomal elements.

## Conclusion

It has long been accepted that lysogenic bacteriophages play an important role in the ability of particular strains of bacteria to cause disease. However, since most pathogens carry one or more lysogenic phage, it is difficult to definitively determine the exact contribution of phage to disease without their precise removal from the genome. This is the first report of a method that systematically cures *S*. *pyogenes* of any and all prophage elements. Our counter–selection technique has allowed us to precisely manipulate lysogeny in the streptococcal genome. The derived KO strains are useful tools to directly examine novel bacteriophage interactions in *S*. *pyogenes* and to elucidate their specific roles in streptococcal survival, pathogenicity, and evolution. The techniques developed in this study could easily be modified for other bacteria-bacteriophage systems, and thus could be beneficial to the study of not just streptococcal-phage interactions but to other areas of research in microbial pathogenesis, ecology or biotechnology.

## Supporting Information

S1 Fig Construction of the counter-selection vector pFWKR. The diagram outlines the steps to construct vector pFWKR. At the top is the location of the *rpsL* gene and upstream promoter region, with adjacent predicted ORFs and their corresponding Spy numbers, from the genome sequence of *S*. *pyogenes* SF370. The bracketed region of DNA was PCR amplified with primers described in the S1 Table and digested with MabI (SibEnzyme, Russia) and EcoICRI (Promega, Madison WI). The amplicon was then ligated into plasmid pFW13, which was previously cut with MabI and SwaI, to create the counter-selection vector pFWKR. The final vector contains the kanamycin resistance gene (aacA/aphD) adjacent to the rpsL^WT^ gene, to make up the Janus cassette. As detailed in the methods section, this counter selection cassette was individually inserted by homologous recombination into each of the prophage of SF370 to screen for mutants that had lost a specific virus. Black arrows and blocks indicate the regions PCR amplified for insertion into the pFW13 vector. The plasmid diagrams indicate relevant restriction sites, multi-cloning sites (MCSI, MCSII),the Janus Cassette: composed of the kanamycin resistance gene (*aacA/aphD*) and the wild type streptomycin sensitive 30S ribosomal protein S12 gene (*rpsL*^WT^) gene, terminators (term) and the *E*. *coli* origin of replication (ori). This figure was designed with the aid of SnapGene® software (from GSL Biotech; available at snapgene.com).
(PDF)

S2 Fig PFGE analysis of *S*. *pyogenes* SF370SmR and 15 permutations of phage deletion mutants. Pulse field gel electrophoresis (PFGE) patterns of SmaI digested genomic DNA from which Fig 2. was derived. Lanes encompass digested DNA from the phage wild type strains SF370 and SF370SmR, as well as all phage KO mutants. Labeled arrows indicate DNA fragments that contain the corresponding integrated phage or phage-like elements, based on the genome sequence of SF370. A loss or drop of these DNA fragments represents a phage or SpyCIM1 deletion. Numbers at top indicate which phages have been deleted (i.e. 1, Φ370.1 (40.9kb); 2, Φ370.2 (42.5 kb); 3, Φ370.3 (33.5Kb); 4, Φ370.4/SpyCIM1 (13.5kb)). λ, Lambda ladder, New England Biolabs PFG Marker with DNA fragment size to right of gel. For a better comparison all phage KO patterns were incorporated into the same figure; lanes corresponding to phage KOs (1,3) and (2,3), bordered by white boxes, were inserted into this figure from a different PFGE gel run under identical conditions. DNA bands in these lanes were aligned to the location of the DNA loading wells at the top, as well as to the four bottom DNA bands in all the lanes, which do not harbor known phage or phage attachment sites.
(PDF)

S3 Fig Plate view of the DNase activity of WT SF370SmR and the full-phage knockout mutant CEM1ΔΦ. Both strains were cultured on DNase Test Agar with Methyl Green for 17 hr. The upper half of the plate contains WT colonies and the lower half of the plate contains colonies from the full phage knockout mutant CEM1ΔΦ. The left side of the plate was inoculated from individual colonies grown on Columbia Blood Agar plates and the right side inoculated from overnight cultures grown in BHI medium. Clearing zones around the colonies are a result of hydrolysis of the DNA and methyl green substrate, signifying DNase activity. This figure includes a color and a black and white photograph of the same plate taken at the same time, to aid in discerning colonies and DNAse activity.
(PDF)

S1 Table Plasmids and primers used in this study. (PDF)
