# Supplementary figures and images for: Targeted Curing of All Lysogenic Bacteriophage from Streptococcus pyogenes Using a Novel Counter-selection Technique
Source: PLoS One. 2016 Jan 12;11(1):e0146408. doi: 10.1371/journal.pone.0146408 (PMC4710455; doi:10.1371/journal.pone.0146408)

S1 Fig.

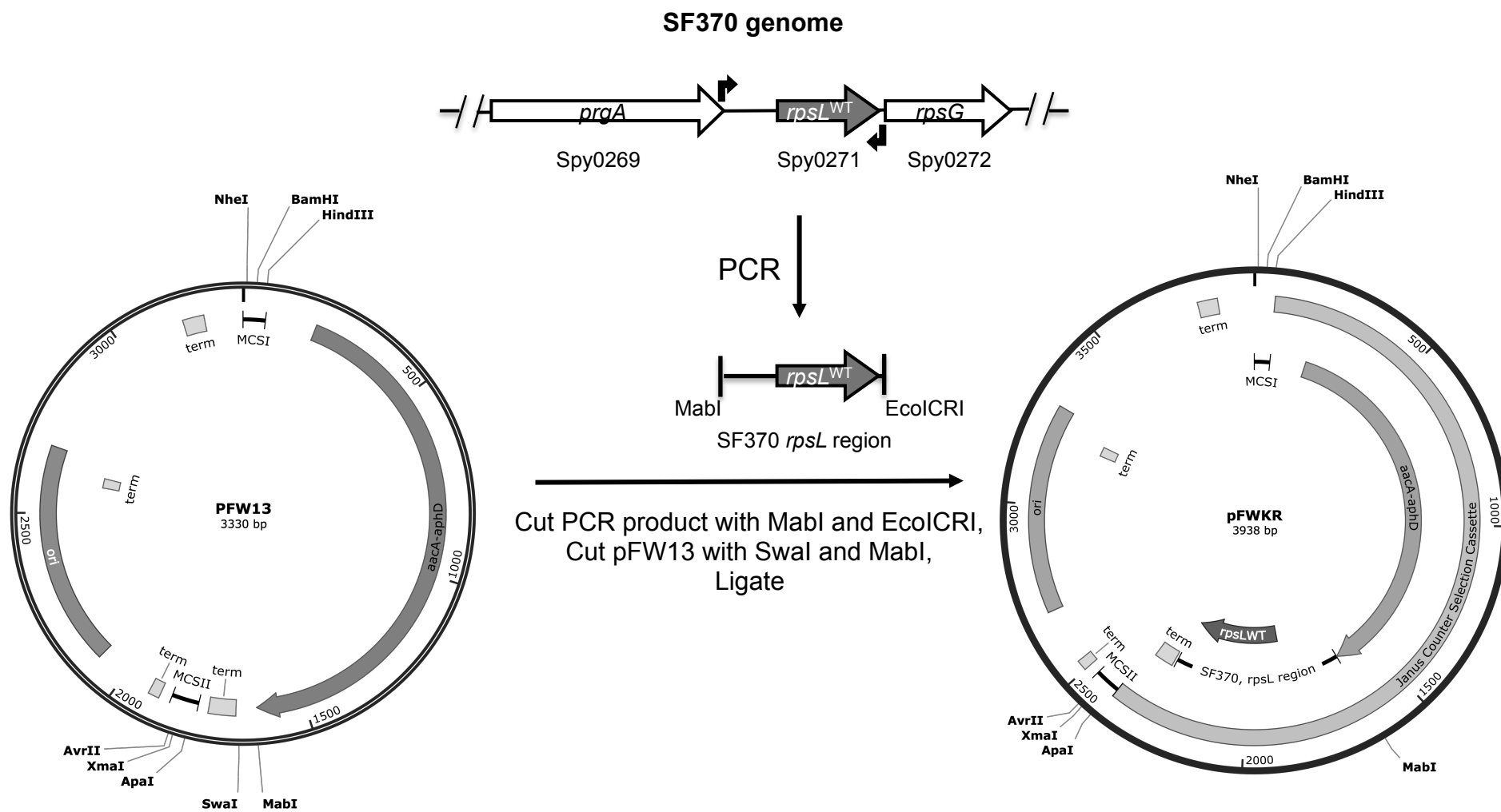

Supplement: S1 Fig — The diagram outlines the steps to construct vector pFWKR. At the top is the location of the rpsL gene and upstream promoter region, with adjacent predicted ORFs and their corresponding Spy numbers, from the genome sequence of S. pyogenes SF370. The bracketed region of DNA was PCR amplified with primers described in the S1 Table and digested with MabI (SibEnzyme, Russia) and EcoICRI (Promega, Madison WI). The amplicon was then ligated into plasmid pFW13, which was previously cut with MabI and SwaI, to create the counter-selection vector pFWKR. The final vector contains the kanamycin resistance gene (aacA/aphD) adjacent to the rpsLWT gene, to make up the Janus cassette. As detailed in the methods section, this counter selection cassette was individually inserted by homologous recombination into each of the prophage of SF370 to screen for mutants that had lost a specific virus. Black arrows and blocks indicate the regions PCR amplified for insertion into the pFW13 vector. The plasmid diagrams indicate relevant restriction sites, multi-cloning sites (MCSI, MCSII),the Janus Cassette: composed of the kanamycin resistance gene (aacA/aphD) and the wild type streptomycin sensitive 30S ribosomal protein S12 gene (rpsLWT) gene, terminators (term) and the E. coli origin of replication (ori). This figure was designed with the aid of SnapGene® software (from GSL Biotech; available at snapgene.com). (PDF) [file pone.0146408.s001.pdf]

S2 Fig.

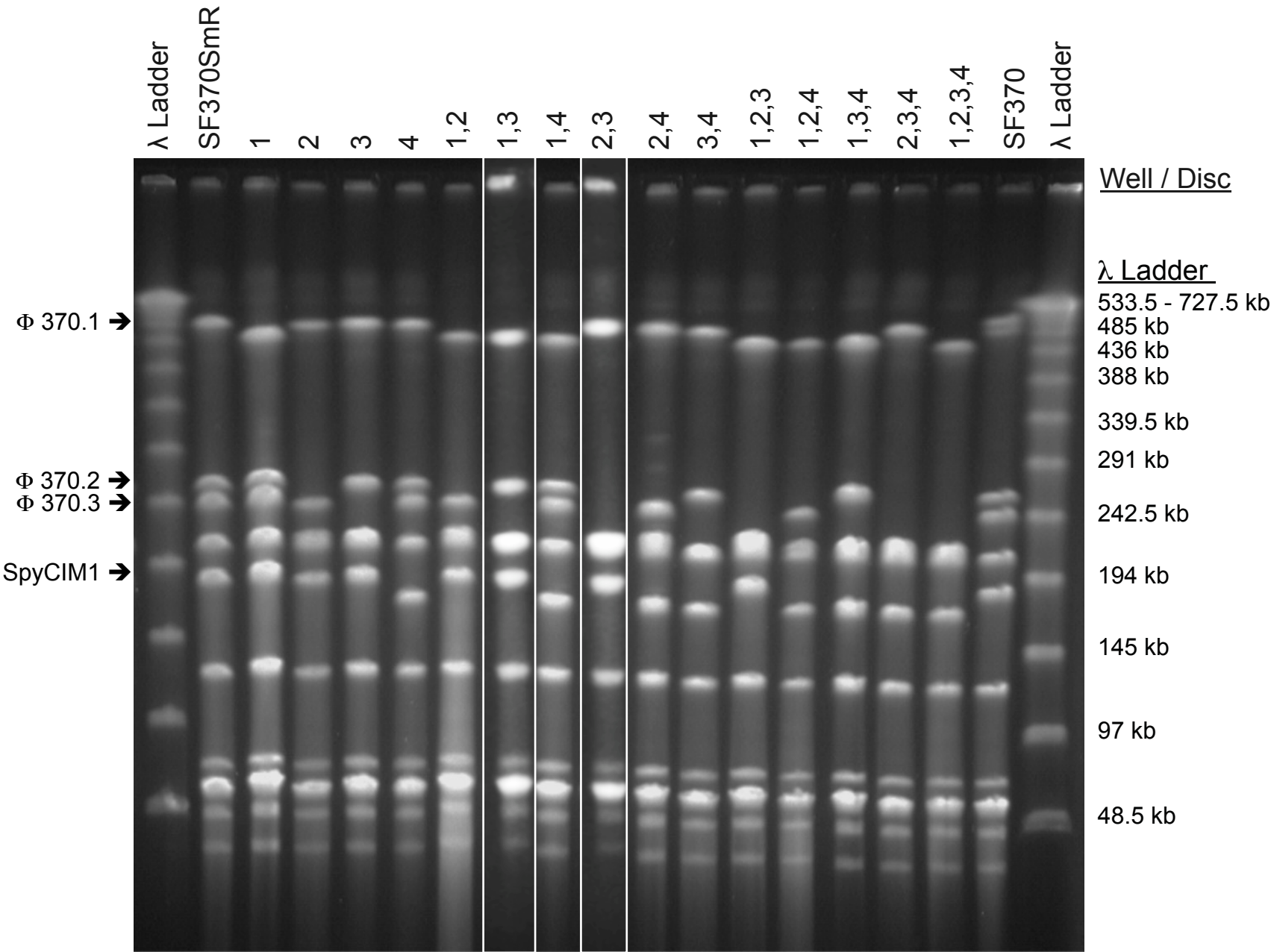

Supplement: S2 Fig — Pulse field gel electrophoresis (PFGE) patterns of SmaI digested genomic DNA from which Fig 2. was derived. Lanes encompass digested DNA from the phage wild type strains SF370 and SF370SmR, as well as all phage KO mutants. Labeled arrows indicate DNA fragments that contain the corresponding integrated phage or phage-like elements, based on the genome sequence of SF370. A loss or drop of these DNA fragments represents a phage or SpyCIM1 deletion. Numbers at top indicate which phages have been deleted (i.e. 1, Φ370.1 (40.9kb); 2, Φ370.2 (42.5 kb); 3, Φ370.3 (33.5Kb); 4, Φ370.4/SpyCIM1 (13.5kb)). λ, Lambda ladder, New England Biolabs PFG Marker with DNA fragment size to right of gel. For a better comparison all phage KO patterns were incorporated into the same figure; lanes corresponding to phage KOs (1,3) and (2,3), bordered by white boxes, were inserted into this figure from a different PFGE gel run under identical conditions. DNA bands in these lanes were aligned to the location of the DNA loading wells at the top, as well as to the four bottom DNA bands in all the lanes, which do not harbor known phage or phage attachment sites. (PDF) [file pone.0146408.s002.pdf]

S3 Fig.

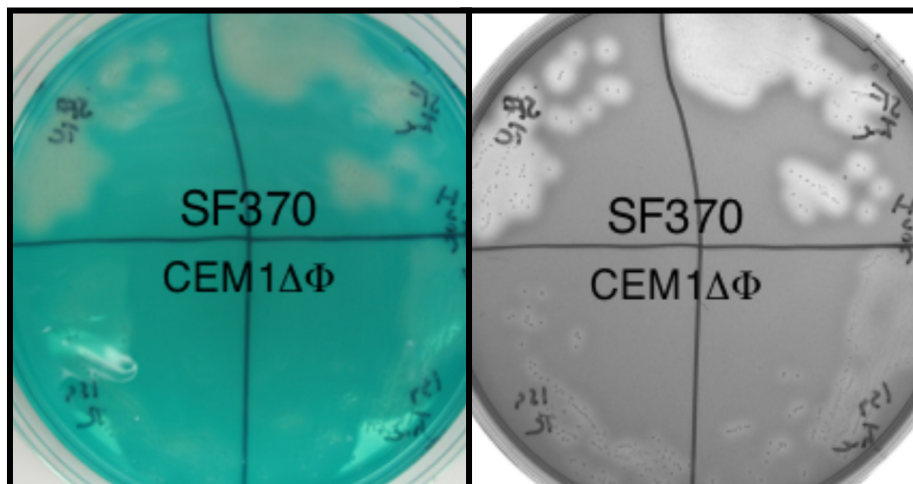

Supplement: S3 Fig — Both strains were cultured on DNase Test Agar with Methyl Green for 17 hr. The upper half of the plate contains WT colonies and the lower half of the plate contains colonies from the full phage knockout mutant CEM1ΔΦ. The left side of the plate was inoculated from individual colonies grown on Columbia Blood Agar plates and the right side inoculated from overnight cultures grown in BHI medium. Clearing zones around the colonies are a result of hydrolysis of the DNA and methyl green substrate, signifying DNase activity. This figure includes a color and a black and white photograph of the same plate taken at the same time, to aid in discerning colonies and DNAse activity. (PDF) [file pone.0146408.s003.pdf]
