# Supplementary material for: Targeted Curing of All Lysogenic Bacteriophage from Streptococcus pyogenes Using a Novel Counter-selection Technique
Source: PLoS One. 2016 Jan 12;11(1):e0146408. doi: 10.1371/journal.pone.0146408 (PMC4710455; doi:10.1371/journal.pone.0146408)
Supplement: S1 Table — (PDF) [file pone.0146408.s004.pdf]

Table S1. Plasmids and primers used in this study

Plasmid or primer	Description [a]	Reference or source[b]
**Plasmids**		
pFW13	*E. coli*-streptococcal shuttle vector; *E. coli* origin of replication and Kan^R gene (*aacA/aphD*)	[52]
pFWKR	Bacteriophage counter-selection vector; pFW13 + SF370 *rpsL*^{WT} (*Sm*^{s}) gene and promoter region inserted after Kan^R gene to create the Phage KO Janus Cassette ((*aacA/aphD*) / *rpsL*^{WT})	This Study
pFWKR-speC	Φ370.1counter-selection vector; pFWKR with adjacent regions to *speC* inserted into MCS sites	This Study
pFWKR-speH	Φ370.2 counter-selection vector; pFWKR with adjacent regions to *speH* inserted into MCS sites	This Study
pFWKR-spd3	Φ370.3 counter-selection vector; pFWKR with adjacent regions to *spd3* inserted into MCS sites	This Study
pFWKR-2136	SpyCIM1counter-selection vector; pFWKR with adjacent regions to *spy2136* inserted into MCS sites	This Study

Primers & Description	Sequence[b,c]	Product size
**Janus Cassette**		
***rpsL*^{WT} gene + upstream promoter from SF370 to clone into pFW13**		**676**
rpsLF1 (MabI)	AGGTTCC**ACCAGGT**ATCTAAGTGATGCATTC	
rpsLR1(EcoICRI)	TTACGACTCAT**GAGCTC**TTATCCCCCTTCTA	
**Janus Cassette from pFWKR for PCR fusion rxns to adjacent regions in each bacteriophage**		**2344**
kan/rpsLFwdFusion	*GCTTGATTTTCGTTCGTGAATACATGTTATAATAAC*	
kan/rpsLRevFusion	*GGCGCGCTTACCAATTAGAATGAATATTTC*	
**Kan^R (*aacA/aphD*) gene from pFw13 (PCR amplicon used as Southern blot probe)**		**1431**
kanFwd	ATGAATATAGTTGAAAATGAAATATGTA	
kanRev	TTAAGTCCTCTTATAAATTTCTTTTCT	
**Φ370.1**		
**Upstream region adjacent to *speC* gene for PCR fusion to Janus Cassette**		**727**
UpSpeCKoFwd-Fus	GACAATAACTGTTGACGCAGCTAGGGTACGAACTTATCC	
UpSpeCKoRev-Fus	*GTTATTATAACATGTATTCACGAACGAAAATCAAGC*TTAGTTTTTAGGAGTGGCAGTTCCATTTAAATAGTCAAGGTTG	
**Downstream region adjacent to *speC* gene for PCR fusion to Janus Cassette**		**588**
DoSpeCKoFwd-Fus	*GAAATATTCATTCTAATTGGTAAGCGCGCC*CATCATACACAAAAAACCGCCCAGAATAATCTGAGC	
DoSpeCKoRev-Fus	CCAATCAGTGCCGTATCACTAATTTGGACATACG	
**Nested PCR of Fusion PCR amplicon for cloning into pFWKR to construct pFWKR-speC**		**3561**
NestNheI370.1 (NheI)	TTGACGCA**GCTAGC**GTACGAACTTATCC	
NestXmaI370.1 (XmaI)	ATTTGGACA**CCCGGG**AATTGTTATCGAG	
**Φ370.1 phage *speC* gene (PCR amplicon also used as Southern blot probe)**		**546**
SpeCFwd	TATAAAAATTGCAGGGTAAA	
SpeCRev	ATAAATATCGAAATGACTAAG	
**Φ370.1 phage *spd1* gene (PCR amplicon also used as Southern blot probe)**		**402**
Spd1/MF2Fwd	CCCCTTCAGGATTGCTGTCAT	
Spd1/MF2Rev	ACTGTTGACGCAGCTAGG	
**Across Φ370.1 phage *attP*-L junction**		**389**
370.1spy0654Fwd	GCTCTTGAGTTCCCAATCACC	
370.1int1Rev	GACTCTCTTGGAATGCTGATGG	
**Across Φ370.1 phage *attP*-R junction**		**1118**
Spd1/MF2Fwd	CCCCTTCAGGATTGCTGTCAT	
370.1PepDRev	AACACGAGAATTGGTAGTGATGG	
**Across Φ370.1 *attB* in phage KO (PCR amplicon also used as Southern blot probe)**		**519**
370.1spy0654F	GCTCTTGAGTTCCCAATCACC	
370.1PepDRev	AACACGAGAATTGGTAGTGATGG	
**Φ370.2**		
**Upstream region adjacent to *speH* gene for cloning into pFWKR to construct pFWKR-speH. From Ryan *et al.* [54]** [b]		**662**
UpSpeHKoFwd (BamHI)	TCTACATA**GGATCC**AACAGAAGTAAAAGG	
UpSpeHKoRev (HindIII)	TTTCTCCTT**AAGCTT**TGAGAGTTTTATTTA	
**Downstream region adjacent to *speH* gene for cloning into pFWKR to construct pFWKR-speH. From Ryan *et al.* [54]** [b]		**888**
DoSpeHKoFwd (ApaI)	CATGTAGAT**GGGCCC**ATCAGCTAATTA	
DoSpeHKoRev (AvrII)	AAAAA**CCTAGG**TATACCAGTCATGACCAGG	
**Φ370.2 phage *speH* gene (PCR amplicon also used as Southern blot probe)**		**318**
SpeHFwd	GTGAATGTCCAGGAAAAGG	
SpeHRev	GCATGCTATTAAAGTCTCCATTG	
**Across Φ370.2 phage *attP*-L junction**		**548**
cpsFQPhageFWDAttL	CTTATGATCACGTAACTGACCGTGC	
inteRevphageAttLRev	GCCCTCAACACCTCTATCAGAC	

**Across Φ370.2 phage attP-R junction**		**753**
HKOFWDW/EXSEQR	CGACTTTGTAACCATTATTGTTCC	
mutXR	GGTCACAGATTTATCCATGAGTTG	
**Across Φ370.2 *attB* in phage KO (PCR amplicon also used as Southern blot probe)**		**906**
cpsFQPhageFWDAttL	CTTATGATCACGTAACTGACCGTGC	
mutXR	GGTCACAGATTTATCCATGAGTTG	
**Φ370.3**		
**Upstream region adjacent to *spd3* gene for PCR fusion to Janus Cassette**		**1058**
UpSpd3KoFwd-Fus	TGAAGATGGTCTGCCATCCATTGTCTGATTGTC	
UpSpd3KoRev-Fus	*GTTATTATAACATGTATTCACGAACGAAAATCAAGC*CTAAACCTTGCCAAGTACGACGATTTGATTTAGACATATCC	
**Downstream region adjacent to *spd3* gene for PCR fusion to Janus Cassette**		**1388**
DoSpd3KoFwd-Fus	*GAAATATTCATTCTAATTGGTAAGCGCGCC*CATAAACTTATACTTAATAAAAATATCTAGATTGTGAACATTATCC	
DoSpd3KoRev-Fus	CGATTGACTTACTAGATAGCGCTGCTGC	
**Nested PCR of Fusion PCR amplicon for cloning into pFWKR to construct pFWKR-spd3**		**4650**
NestXmaI370.3 (XmaI)	TTTAGTT**CCCGGG**CAATTAAAAGTTATTC	
NestNheI370.3 (NheI)	GAAGTCCACC**GCTAGC**CGACAG	
**Φ370.3 phage *spd3/mf3* gene (PCR amplicon also used as Southern blot probe)**		**482**
spd3/MF3Fwd	CAAATTGACTGACGCTAATGG	
spd3/MF3Rev	CCGCTTCTTCAAACTCTTCG	
**Across Φ370.3 phage *attP*-L junction**		**875**
Spy1434out370.3JxnF	TCTTCACCAAATCCAATATACCC	
370.3ATTLjxntestR	CTAACATACGACGAGTTTAAGC	
**Across Φ370.3 phage *attP*-R junction**		**760**
hlpASF370.3JXR	ATGGCTAACAAACAAGATTTAATCG	
int2SF370.3F	GACATTAGCTTCTTATCGTGAAACC	
**Across Φ370.3 *attB* in Phage KO (PCR amplicon also used as Southern blot probe)**		**806**
Spy1434out370.3JxnF	TCTTCACCAAATCCAATATACCC	
hlpASF370.3JXR	ATGGCTAACAAACAAGATTTAATCG	
**SpyCIM1**		
**Upstream region adjacent to *spy2136* gene for PCR fusion to Janus Cassette**		**781**
UpSpy2136KoFwd-Fus	CAGTTTAGACCTTTGAAACCGCCTGAGG	
UpSpy2136KoRev-Fus	*GTTATTATAACATGTATTCACGAACGAAAATCAAGC*TGCTGG TAAACGTCCTATCTATCTCATCAATGG	
**Downstream region adjacent to *spy2136* gene for PCR fusion to Janus Cassette**		**818**
DoSpy2136KoFwd-Fus	*GAAATATTCATTCTAATTGGTAAGCGCGCC*TTAGTCATTTTATACGTTTTAGCATTTAAAAAATTAAACG	
DoSpy2136KoRev-Fus	GTGATAAGTTGCGTCAATCACTGGTTGC	
**Nested PCR of Fusion PCR amplicon for cloning into pFWKR to construct pFWKR-spy2136**		**3842**
NestNheISpyCIM1 (NheI)	TACCAGTCA**GCTAGC**CTAAAATAGCATGACAAGC	
NestXmaISpyCIM1 (XmaI)	GAACACAAG**CCCGGG**ACTGCCTAAGT	
**SpyCIM1 Primase gene *spy2136* (PCR amplicon also used as Southern blot probe)**		**950**
SPy2136PrimaseFwd	TTGCTA AAGCAGAATACAGAAAGAGAGG	
SPy2136PrimaseRev	ATTACAATGGATTTCCCTAGAAGTATCG	
**SpyCIM1 Integrase (PCR amplicon also used as Southern blot probe)**		**564**
SpyCIM1intFwd	TAAAGCGTTCAATCCCTGCT	
SpyCIM1intRev	CTGCGGTACGTTCAGTCATC	
**Across SpyCIM1 *attP*-L junction**		**919**
mutS-MutLattBFwd	TCGCTGGCCTTTCTACAACT	
SpyCIM1intRev	CTGCGGTACGTTCAGTCATC	
**Across SpyCIM1 *attP*-R junction**		**429**
SpyCIM1attRFwd	TACCCTGTAAAGACTGCC	
mutS-MutLattBRev	CAGGACTGCCAAAATCCCTA	
**Across SpyCIM1 *attB* in phage KO (PCR amplicon also used as Southern blot probe). From Scott *et al.* [13][b]**		**461**
mutS-MutLattBFwd	TCGCTGGCCTTTCTACAACT	
mutS-MutLattBRev	CAGGACTGCCAAAATCCCTA	

[a] Abbreviations used: Kan^R, kanamycin-resistant; Sm^S, streptomycin-sensitive; MCS, multiple cloning site.

[b] All plasmids and primers were derived from this study unless otherwise stated:

[c] All sequences are 5' to 3'. Restriction sites engineered into each primer are denoted in bold and the corresponding restriction enzymes are indicated in parentheses. Italicized text indicates overlapping primer sequences used for PCR based fusion and assembly of the Janus Cassette between the adjacent regions of phage genes targeted for allelic replacement.
